# High-resolution deep sequencing reveals biodiversity, population structure, and persistence of HIV-1 quasispecies within host ecosystems

**DOI:** 10.1186/1742-4690-9-108

**Published:** 2012-12-17

**Authors:** Li Yin, Li Liu, Yijun Sun, Wei Hou, Amanda C Lowe, Brent P Gardner, Marco Salemi, Wilton B Williams, William G Farmerie, John W Sleasman, Maureen M Goodenow

**Affiliations:** 1 Department of Pathology, Immunology and Laboratory Medicine, College of Medicine, University of Florida, 2033 Mowry Road, PO Box 103633, Gainesville, FL, 32610-3633, USA; 2 Interdisciplinary Center for Biotechnology Research, University of Florida, Gainesville, FL, USA; 3 Department of Epidemiology and Health Policy Research, College of Medicine and Department of Biostatistics, College of Public Health, University of Florida, Gainesville, FL, USA; 4 Department of Pediatrics, Division of Allergy, Immunology and Rheumatology, College of Medicine, University of South Florida, and All Children’s Hospital, St. Petersburg, FL, USA

**Keywords:** HIV-1 envelope V3, Biodiversity, Population structure, Quasispecies, Fitness, Pyrosequencing, Founder virus persistence, Most recent common ancestor

## Abstract

**Background:**

Deep sequencing provides the basis for analysis of biodiversity of taxonomically similar organisms in an environment. While extensively applied to microbiome studies, population genetics studies of viruses are limited. To define the scope of HIV-1 population biodiversity within infected individuals, a suite of phylogenetic and population genetic algorithms was applied to HIV-1 envelope hypervariable domain 3 (Env V3) within peripheral blood mononuclear cells from a group of perinatally HIV-1 subtype B infected, therapy-naïve children.

**Results:**

Biodiversity of HIV-1 Env V3 quasispecies ranged from about 70 to 270 unique sequence clusters across individuals. Viral population structure was organized into a limited number of clusters that included the dominant variants combined with multiple clusters of low frequency variants. Next generation viral quasispecies evolved from low frequency variants at earlier time points through multiple non-synonymous changes in lineages within the evolutionary landscape. Minor V3 variants detected as long as four years after infection co-localized in phylogenetic reconstructions with early transmitting viruses or with subsequent plasma virus circulating two years later.

**Conclusions:**

Deep sequencing defines HIV-1 population complexity and structure, reveals the ebb and flow of dominant and rare viral variants in the host ecosystem, and identifies an evolutionary record of low-frequency cell-associated viral V3 variants that persist for years. Bioinformatics pipeline developed for HIV-1 can be applied for biodiversity studies of virome populations in human, animal, or plant ecosystems.

## Background

Human immunodeficiency virus type 1 (HIV-1) displays extensive genetic diversity, reflecting the error prone characteristics of reverse transcriptase-dependent replication, elevated recombination rate and continuous selection of more fit viral variants within fluctuating host ecosystems. HIV-1 populations within an infected individual are complex and comprised of swarms of related genomes, or quasispecies [1,2]. Studies of HIV-1 diversity within quasispecies benefited over the years by the development of novel sequencing technologies that extended the depth of sampling [1-11]. Next generation deep sequencing increases significantly the sensitivity to identify within HIV-1 quasispecies low frequency genetic variants that might lead to reduced susceptibility to antiretroviral treatments [12,13] or escape from immunity [14]. Beyond surveillance for drug resistance, deep sequencing provides additional advantages to detect epistatic interactions [15], estimate population structure [16], identify evolutionary intermediates, and evaluate biodiversity of organisms within an ecosystem [17-26].

Biodiversity is used in population genetics to present a unified view of the extent of variation of life forms within habitats [27] and assumes that genomes within an environment are taxonomically similar, randomly distributed, and sufficiently large [28]. Assessments of biodiversity from deep sequencing data provide unprecedented views of the richness of immune loci in primates, zebra fish, and humans [17,18,26] or the complexity of microbiomes independent of an ability to culture microorganisms [21,24,25,29]. Biodiversity defines complexity within populations that extend beyond evaluations of diversity based on pairwise genetic distance, the major approach for analysis of small data sets of HIV-1 sequences from infected individuals [30,31]. Biodiversity within HIV-1 populations might reflect host environments, infection by circulating recombinant forms of HIV-1 or co-infection by multiple subtypes, and provide unique and sensitive biomarkers for changes in viral populations. Moreover, structure of HIV-1 quasispecies, or the frequency distribution of viral variants within individuals, may reveal the potential for viral populations to evolve within a fitness landscape and contribute to viral persistence [4,32-34].

We designed a deep-sequencing study of HIV-1 Env V3 quasispecies within peripheral blood cells that applied population genetics tools in a novel bioinformatics pipeline to define viral biodiversity, examine viral population structure, and explore directly the extent to which deep sequencing enriches analysis of the HIV-1 evolutionary landscape.

## Results

### Biodiversity of HIV-1 quasispecies

**Figure 1:**
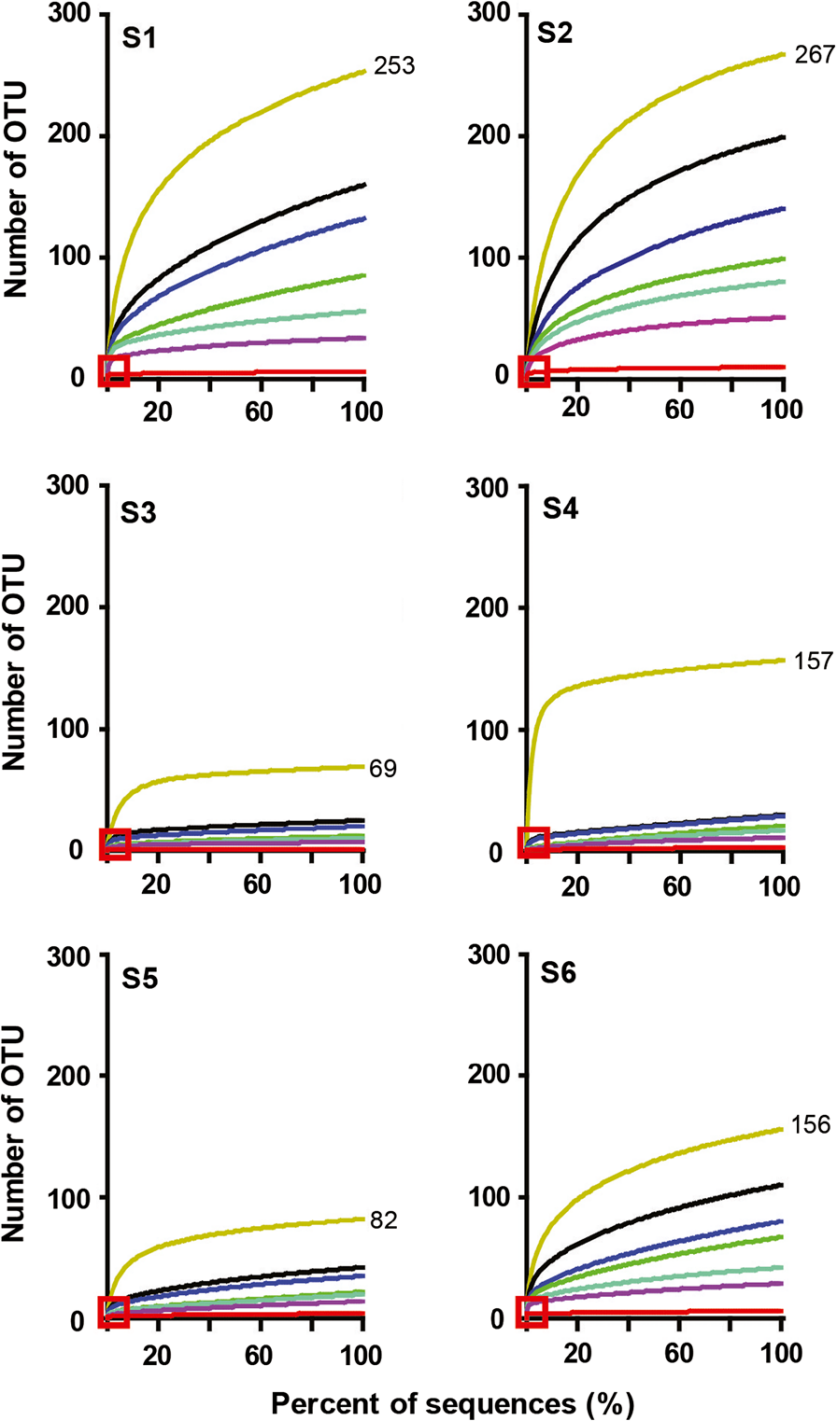
**Biodiversity among viral populations.** Pyrosequencing data sets from each individual were clustered at 0% (unique) to 10% genetic distances and displayed as rarefaction curves. Y-axis, number of OTU (number of sequence clusters); x-axis, percent of total pyrosequences (sequences sampled ÷ total number of sequences x 100%). Colors of curves indicate the level of clustering: yellow, 0%; black, 1%; blue, 2%; green, 3%; cyan, 4%; purple, 5%; red, 10%. Numbers of OTU at the end of curves at 0% distance represent biodiversity calculated from rarefaction curve at the sequence depth (100% of pyrosequences). Small red boxes indicate approximate sequence depth achieved by conventional clonal sequences.

Biodiversity is evaluated by rarefaction analysis and defined as the number of operational taxonomic units (OTU) within a population [17,18,21,23-26]. HIV-1 Env V3 pyrosequences within each sample were clustered over a range of pairwise genetic distances from 0% to 10% to compare viral populations among individuals (Figure 1). When an upper clustering threshold of 10% was applied to approximate mean pairwise genetic distance found among subtype B Env sequences [35], the virus population formed a single OTU in S3, but included 3 or 4 OTU in S4 or S5 viral populations, 6 OTU in S1 and S6, or as many as 10 OTU in S2 (Table 1). Biodiversity of viral populations evaluated at 0% distance (i.e., the unique level) [31] ranged from relatively low biodiversity (69 or 82 OTU) in S3 or S5, to 156 or 157 OTU in S6 or S4, or as high as 253 or 267 OTU in S1 or S2 (Figure 1). Even though viral biodiversity at the unique level was similar between some individuals, clustering genomes at distances from 1% to 5% revealed differences in complexity within host environments. For example, the virus population in S4 displayed reduced complexity compared with the population in S6, and was more similar to viral populations in S3 or S5 (Figure 1 and Table 1). Biodiversity calculated at 3% correlated significantly with biodiversity at the unique level among the individuals [r = 0.91, p = 0.01] and provided a rationale for clustering at 3% in subsequent analyses.

**Table 1:** Calculated and estimated biodiversity defined by operational taxonomic units (OTUs)

**PID**^**a**^	**Biodiversity (OTU)**^**b**^					
	**0%**		**3%**		**10%**	
	**Calculated**	**Estimated**	**Calculated**	**Estimated**	**Calculated**	**Estimated**
S1	253	315	85	178	6	7
S2	267	293	99	137	10	12
S3	69	75	12	24	1	1
S4	157	183	21	55	3	4
S5	82	98	22	91	4	5
S6	156	200	67	132	6	7

^a^ Patient identification.

^b^ Biodiversity, expressed as operational taxonomic units (OTU), was calculated from rarefaction curves or estimated by abundance-based estimator (ACE) using ESPRIT software [25] when sequences were clustered at 0% (unique), 3%, or 10% distance.

Rarefaction curves at 3% distance approached, but failed to achieve a plateau, raising the possibility that depth of sequencing was insufficient to capture all viral diversity. Yet, estimated maximum biodiversity was only about two-fold greater than, and correlated with, calculated biodiversity (r = 0.89; p = 0.02) (Table 1), indicating that sequence depth (about 25-fold coverage) was sufficient to provide a robust assessment of V3 biodiversity within a sample. In general, biodiversity among the six subjects appeared unrelated to viral levels in plasma or cells, length of infection, or CD4 T cell levels (Additional file 1), but revealed patterns of complexity within viral quasispecies in different host environments.

### Population structure

**Figure 2:**
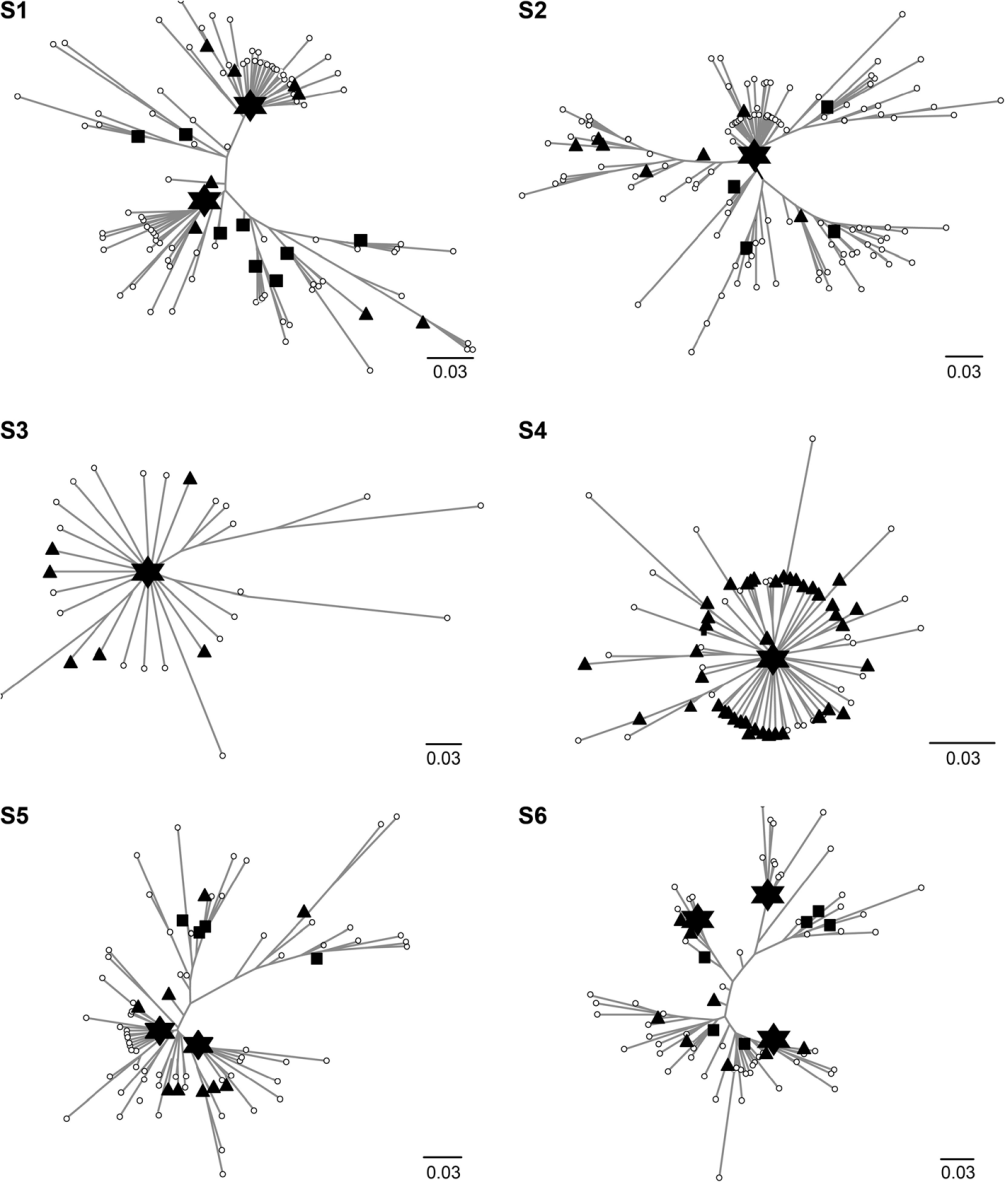
**Organization of viral populations.** Unrooted neighbor-joining trees were developed for each pyrosequencing data set clustered at 3% pairwise distance. Symbols represent the proportion of total pyrosequences in a cluster: *empty circle*, ≤ 0.25%; *black inverted triangle*, > 0.25% to 1%; *black square*, > 1% to 10%; *star*, > 10%.

To evaluate the complexity of viral population structure within each individual, unrooted phylogenetic trees were constructed to relate the distribution and frequency of sequence clusters (OTUs) with the proportion of amino acid sequences in each cluster. Each subject harbored virus populations in which 65% to 90% of sequences were organized into 1 to 3 dominant clusters with thousands of sequences per cluster (Figure 2). In each case, dominant sequence clusters were surrounded by swarms of clusters of less abundant variants forming star-like phylogenies. In general, structure of viral populations in different environments was distinguished not only by the number of dominant sequences, but by the distribution of the frequency of non-dominant viral variants, as well; for example, viruses in S2, S3, and S4 each had a single dominant population, but unique organization and frequency of less abundant variants.

### Enriched evolutionary landscape within HIV-1 quasispecies

**Figure 3:**
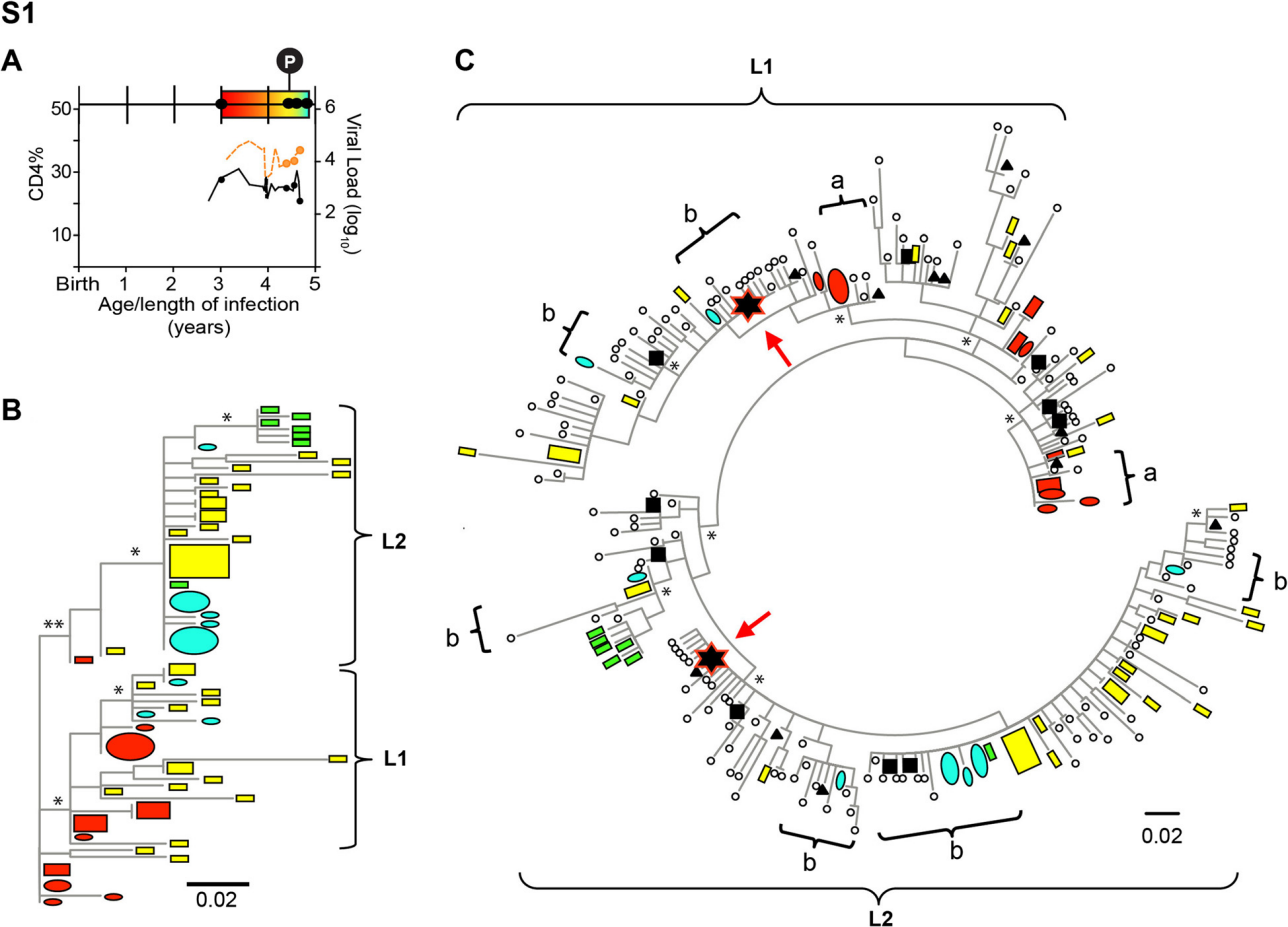
**Persistence of V3 variants in PBMC for S1.A**. Time line with rainbow colors indicate timing of samples (black dots for clonal sequences; black dot with P for pyrosequences), as well as CD4% (black line) and log_10_ plasma viral levels (orange line), relative to age/length of infection in years. **B**. ML tree of longitudinal clonal V3 sequences resembled the topology of trees developed from Env V1 through V3 clonal sequences (sequence number: red – 19, yellow – 37, green – 7, blue - 13). Symbols: ovals, plasma RNA sequences; rectangles, cell-associated DNA sequences. Size of symbols represents relative abundance of sequences in the population. Colors represent time line of samples. Asterisks on a branch represent significant approximate likelihood-ratio test (* > 0.75, ** >0.90). Scale indicates 0.02 nucleotide substitutions per site. **C**. ML tree combining longitudinal conventional and single-time point deep sequences. Black symbols represent pyrosequences clustered at 3% pairwise distance with symbol shapes indicating proportion of sequences in each cluster: *empty circle* ≤ 0.25%; *black inverted triangle*, > 0.25% to 1%; black square, > 1% to 10%; *star*, > 10%. Brackets indicate colocalization of cell-associated viral variants by pyrosequencing with: “a”, clonal RNA and DNA viral sequences from earlier time point; or “b”, clonal plasma viral variants from later time points. Scale indicates 0.02 nucleotide substitutions per site.

To evaluate the relationship of archived viral populations from a single time point to viral populations over time, phylogenetic trees were inferred from deep sequence V3 data sets combined with longitudinal cell-associated and plasma clonal viral sequences. Combined data sets from S1 extended over a two-year period from about 3 to 5 years of age/infection, when CD4 T cells ranged between 25% to 30% and viral set point was about 10,000 copies (Figure 3A). Phylogenetic analysis of conventional clonal V3 sequences from viral DNA and RNA at four time points provided a view of viral populations with significantly supported branches (L1 and L2), but unclear dominant viral population(s) (Figure 3B). When pyrosequencing data were included in the phylogenetic construction, two dominant populations, one in L1 and the second in L2, became apparent (Figure 3C). Low frequency (~1%) cell-associated V3 pyrosequencing variants colocalized on the tree with virus found about eighteen months earlier by conventional sequences in both cells and plasma. Moreover, pyrosequencing variants with frequency ranging from 0.25% to >10% in cells colocalized with conventional sequences found months later in plasma viral RNA. Overall, the array of viral variants identified by pyrosequencing at a single time point reflected the range of clonal sequences identified in longitudinal samples over 2-years of infection.

**Figure 4:**
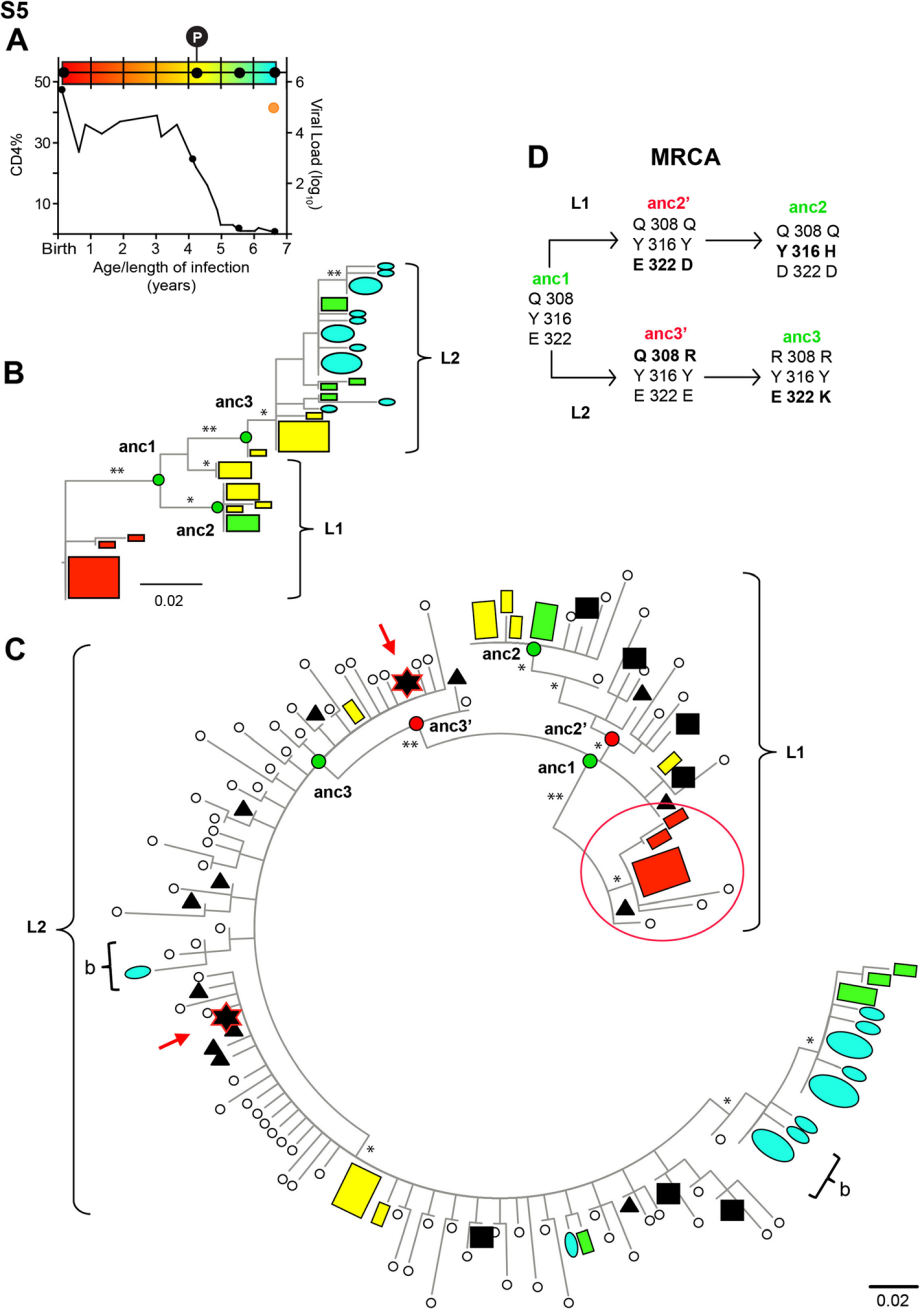
**Persistence of V3 variants and evolutionary intermediates.A**. Time line with rainbow colors indicates timing of samples (black dots, clonal sequences; P, pyrosequences), CD4% (black line) and log_10_ plasma viral load at one time point (an orange dot), relative to age/length of infection in years. **B**. ML tree of conventional sequences (sequence number: red – 10, yellow – 15, green – 8, blue - 17) with most recent common ancestral nodes (anc) labeled for different lineages (green circles). Scale: 0.02 nucleotide substitutions/site. Symbols: ovals, plasma RNA sequences; rectangles, cell-associated DNA sequences. Size of symbols: relative abundance of sequences in the population. Colors: timing of samples. Asterisks on branches: significant approximate likelihood-ratio test (* >0.75, ** >0.90). **C**. ML tree combining longitudinal conventional and single-time point pyrosequences with anc nodes marked for different lineages (green circles: the same anc nodes as in panel B; red circles: additional anc nodes when pyrosequences filled in the phylogenetic landscape). Black symbols: represent pyrosequences clustered at 3% distance with symbol shapes indicating proportion of sequences in each cluster: *empty circle* ≤ 0.25%; *black inverted triangle*, > 0.25% to 1%; *black square*, > 1% to 10%; *star*, > 10%. Brackets with “b”: clustering of cell associated viral variants by pyrosequencing with clonal plasma viral variants from a later time point. Red circle: colocalization of cell-associated virus from near birth with a subset of pyrosequences in cells 4.5 years later. **D**. Most recent common ancestors (MRCA) on ML tree of panel C. Anc1, anc2 and anc3: the same ancestral nodes on ML tree in panel B. Anc2’ and anc3’: additional ancestral nodes when pyrosequences fill in the evolutionary landscape. Numbers: amino acid positions relative to HIV-1_HXB2_ gp160 [36]. NOTE. MRCA analysis was not performed on S1 data because only single amino acid changes occurred between ancestral nodes on the conventional ML tree.

To evaluate viral populations over longer periods of time, S5 samples collected from 6-wks to more than 6.6 years of age were analyzed (Figure 4A). Cell-associated V3 variants by conventional clonal sequencing shortly after birth had limited diversity, while at least two well-supported lineages of variants (L1 and L2) developed by 4.4 years of infection (Figure 4B). Pyrosequences included two dominant clusters, both in L2, as well as the repertoire of V3 domains found over the course of infection (Figure 4C). For example, some low frequency (~1%) cell-associated virus quasispecies found after 4.5 years of infection included V3 domains that colocalized with the cluster of viral DNA sequences identified shortly after birth (Figure 4C). Other low frequency cell-associated V3 variants detected by pyrosequencing (0.25%) were closely related to viral RNA expressed in plasma more than two years later. Overall, the evolutionary landscape was defined by cyclic emergence of dominant populations from low-frequency variants.

### Most recent common ancestors in the evolutionary landscape

V3 populations in S5 developed along lineages with multiple amino acid changes at branch nodes, providing an opportunity to infer the most recent common ancestor (MRCA) of each lineage. Based on clonal sequences, the earliest viral population gave rise through ancestral node 1 (anc1) to two subsequent lineages (Figure 4B). L1 progressed through node 2 (anc2) with changes in V3 at two amino acid positions, E322D and Y316H (Figure 4D), while L2 gave rise by two different amino acid substitutions, Q308R and E322K (Figure 4D) to viruses at 6 to 7 years of infection through anc3 (Figure 4B). Depth of conventional clonal sequencing was inadequate to assign a temporal order to the amino acid changes between MRCA at anc1 and anc2 or anc3. Inclusion of pyrosequences in the analysis provided sufficient coverage of the viral population to infer that the E322D change (anc2’) appeared before the Y316H substitution, while Q308R (anc3’) preceded the E322K substitution (Figure 4D).

## Discussion

Biodiversity is routinely applied to metagenomics of a variety of species, including the human microbiome, but only limited, if any, assessment of viromes in different ecological niches. Our study applies an efficient bioinformatic pipeline that we developed to assess the complexity of HIV-1 quasispecies in unique ecosystems within infected individuals. The power of pyrosequencing to generate extensive sequence data sets provides a foundation to apply population genetic analyses and extends the value for deep sequencing beyond analysis of rare variants that might indicate reduced sensitivity to drugs. Analysis of biodiversity based on sequence clustering provides a novel viral population profile for different environments independent of viral levels in cells or plasma, perhaps reflecting length of infection if sequences were archived in lineages of long-lived cells. Consistent with this model, complex viral population structure with high biodiversity appeared as early as eighteen months, or by four to six years, of infection in some individuals. Yet, similar periods of infection in other individuals were characterized by monomorphic viral populations with low complexity, indicating that biodiversity of V3 populations represents complex combinations of factors; for example, changes in viral fitness in the environmental landscape in response to host immunity, host target cells, or coreceptor evolution under selective pressure.

Another novel aspect of our study involved a combination of cross-sectional deep sequencing with conventional longitudinal sequences to provide high-resolution detection of evolutionary intermediates, which may be less fit or infrequent in peripheral blood, but nonetheless contribute to the genetic flexibility of the population. The specific order of amino acid substitutions over time may reflect important epistatic interactions that could focus detection of compensatory mutations contributing to fitness in the genetic landscape to other regions of the virus genome. Deep sequencing data sets fill in the evolutionary landscape and increase the power to infer the temporal accumulation of amino acid substitutions, or provide a basis for rational functional analysis of ancestral envelopes and the progeny that emerge from recurring viral population bottlenecks.

An apparent paradox from our analyses is the contribution by low-frequency, presumably less-fit viral variants, rather than the dominant variants, to next generation plasma HIV-1 populations with enhanced fitness. Low-frequency variants expand the fitness landscape for virus populations, while providing an array of evolutionary options to maximize survival in a changing ecosystem [34]. Low frequency cell-associated HIV-1 quasispecies may represent residual genomes from a past dominant population archived in long-lived cells, a sequestered reservoir that only infrequently finds its way into the peripheral blood, and/or progenitors that gives rise to the next generation of dominant variants in the plasma. Transient dominance of a population leaves a molecular trail that persists as low frequency variants archived in peripheral blood. In agreement with studies of heterosexual HIV-1 transmission [37], archeological evidence of the earliest viral populations was found in our study of pediatric cells as long as four years after infection by maternal transmission, suggesting those early viruses, or at least their V3 domains, endure during the natural history of infection.

While the study focused on HIV-1 populations in human environments, the approach is applicable to an array of viruses with complex populations, including other subtypes or recombinant forms of HIV-1, hepatitis C or hepatitis B viruses, as well as the repertoire of related viruses that infect animals. Increased depth of sampling and extended length of the target region now possible by pyrosequencing combined with efficient bioinformatic pipelines provides a basis for developing quantitative measures of the ebb and flow of viral populations in changing environments.

## Conclusions

Deep sequencing of HIV-1 Env V3 hypervariable domains combined with conventional longitudinal V3 sequence data sets provides high resolution of the evolutionary landscape of HIV-1 quasispecies, reveals the richness of viral diversity within the ecosystems of infected individuals, explores the ebb and flow of dominant high-fit and low frequency less-fit viral variants, infers details of multistep evolutionary events in the fitness landscape, and identifies persistence of low-frequency viral variants in peripheral blood cells that resemble transmitted viruses.

## Methods

### Subjects

Peripheral mononuclear cells (PBMC) were obtained from a cohort of HIV-1 children with parental informed consent under a protocol approved by the Institutional Review Board of the University of Florida. Study included six therapy-naïve subjects, infected perinatally between 1989 and 1995 through maternal transmission of subtype B HIV-1, with median plasma viral load of 4.9 (quartile range 4.6 to 5.3) log_10_ HIV-1 RNA copies per ml, median age/length of infection of 4.4 (quartile range: 2.0 to 5.1) years, and median CD4 levels of 22% (quartile range 13.3% to 25.5%) at the time of deep sequencing (Additional file 1).

### Clonal and pyrosequences

Clonal sequences from HIV-1 Env V1 through V5 were generated using AmpliTaq (Life Technologies Corporation, Carlsbad, CA, US) as previously described [30]. Amplicon libraries were constructed from PBMC DNA with 400 HIV-1 copies using GoTaq DNA polymerase (Promega, Madison, WI, US), as previously described [38,39] and submitted to the University of Florida Interdisciplinary Center for Biotechnology Research for pyrosequencing using a proprietary DNA polymerase (a mixture of *Taq* and high fidelity DNA polymerases) (Roche/454 Life Sciences) on a Genome Sequencer FLX (Roche/454 Life Sciences) to produce an average of about 10,000 reads per sample or about 25-fold coverage of 400 template copies (10,000 sequences ÷ 400 viral copies = 25 fold coverage). Raw clonal and pyrosequencing nucleic acid data sets are deposited in EMBL data base (EMBL accession numbers pending).

### Analysis pipeline

A bioinformatics pipeline developed by our group was applied to the data sets. The pipeline incorporates a series of quality control and error correction filters to reduce random nucleotide substitutions, correct frame shifts, and eliminate hypermutated or recombinant sequences (Additional file 2). Overall, the analysis pipeline produced high-quality data sets with retention of about 90% to 97% of the sequences from any sample (Additional file 3). Integrity of error-corrected datasets from deep sequencing was verified by phylogentic construction (Additional file 4).

In general, maximum likelihood pairwise distances within deep sequence data sets were significantly greater than among conventional sequence data from each individual (p < 0.001). To assess biodiversity of HIV-1 Env quasispecies, rarefaction curves were constructed using the ESPRIT software suite [25]. Numbers of OTU are displayed on the y-axis as a function of percentage of sequences (sequences sampled ÷ total sequences generated from 400 input viral copies x 100%) displayed on the x-axis. Sequences were clustered across a range of pairwise distances from 0% to 10% with all previously collapsed reads counted for their absolute occurrence. One OTU equates to one sequence cluster. ESPRIT was also used to estimate maximum biodiversity within 400 input viral copies using abundance-based coverage estimator (ACE), constructed consensus sequence from each sequence cluster, and calculated the frequency of each OTU.

### Construction of phylogenetic trees and most recent common ancestor (MRCA) analysis

Maximum likelihood (ML) phylogenetic trees combined deep sequencing cluster consensus reads and longitudinal clonal sequences for subjects S1 and S5 were constructed from nucleotide sequences aligned in BioEdit. Alignments were trimmed to the V3 loop defined by codons for cysteine 296 to cysteine 331 based on gp160 amino acid numbering in HXB2 genome, and identical nucleic acid clusters were collapsed.

Phylogenetic signal within S1 or S5 datasets of aligned sequences was evaluated by likelihood mapping analyses with the program TREE-PUZZLE, and proven to be sufficient for reliable phylogeny inference [40-42] (Additional file 5). Trees were constructed as previously described [9]. Briefly, the heuristic search for the best tree was performed using a neighbor-joining tree and the tree bisection reconnection algorithm with PAUP* 4.0b10 [43,44]. Trees were rooted using the earliest clonal sequences as the out group. Significance of branches was determined by the approximate likelihood ratio test [45-47]. For analysis of MRCA, ancestral nucleic acid sequences in the genealogy obtained for S5 were inferred by the maximum likelihood method using the codon substitution model M0 in the PAML software package [47]. Reconstructed ancestral sequences from internal nodes were analyzed in BioEdit for nonsynonymous changes at each codon position.

### Statistical analysis

Pearson correlation was applied to analyze correlations between biodiversity calculated from rarefaction curves generated at 0% and 3% pairwise distances, and between calculated and ACE-estimated maximum biodiversity. Statistical analyses were performed using SAS version 9.1 (SAS 191 Institute, Cary, NC) with P < 0.05 defined as significant.

## Competing interests

The authors declare that they have no competing interests.

## Authors’ contributions

LY, WGF, JWS, and MMG designed the study, obtained funding, analyzed and interpreted the results. JWS directed the clinical program and provided clinical samples and data about the subjects. LY and LL with WGF, YS, and MMG were involved in developing analytical pipeline for data analysis, and applying population genetics analysis tools. LY developed the experiments, the methods, and supervised data acquisition and analysis by BPG, WBW, and YS, and collaborated with WH for biostatistical analyses; MMG worked with ACL and MS to analyze distances, phylogeny, most recent common ancestor, and integration of deep sequencing with conventional sequences. Manuscript was written by LY and MMG with input from all authors. All authors read and approved the final manuscript.

## Authors’ information

LL is currently a faculty member at the University of Arizona.

YS is currently a faculty member at the University of Buffalo.

WH is currently a faculty member at the Stony Brook University Medical Center.

BPG is currently a medical student in Philadelphia College of Osteopathic Medicine in Suwanee, Georgia. WBW is currently a postdoctoral research fellow at the Duke University.

## Supplementary Material

Additional file 1 **Table S1.** Characteristics of study participants at time of pyrosequencing. Click here for file

Additional file 2 Error correction. Click here for file

Additional file 3 **Table S2.** Sequential filtering of data sets through the bioinformatics pipeline. Click here for file

Additional file 4 **Figure S1.** Phylogenetic tree of clustered error-corrected pyrosequences from individuals studied. Click here for file

Additional file 5 **Figure S2.** Likelihood mapping analysis to evaluate phylogenetic signal. Click here for file
