# Supplementary material for: High-resolution deep sequencing reveals biodiversity, population structure, and persistence of HIV-1 quasispecies within host ecosystems
Source: Retrovirology. 2012 Dec 17;9:108. doi: 10.1186/1742-4690-9-108 (PMC3531307; doi:10.1186/1742-4690-9-108)
Supplement: Additional file 1 — Table S1. Characteristics of study participants at time of pyrosequencing. [file 1742-4690-9-108-S1.pdf]

Additional materials

Additional file 1: Table S1. Characteristics of study participants at time of pyrosequencing.

**PID**[a]	**Viral load**		**Age/ length of infection (years)**	**CD4 %**[d]
	**Plasma**[b]	**Cell-associated**[c]		
**S1**	4.0	2.6	4.5	24
**S2**	4.8	4.5	1.5	30
**S3**	4.9	3.4	4.8	22
**S4**	4.9	3.3	2.1	17
**S5**	5.2	5.0	4.2	22
**S6**	5.7	5.5	6.1	2

[a] Patient identification. Subjects included in pyrosequencing study were infected perinatally by maternal transmission for more than one year, were naïve to combination antiretroviral therapy with viral RNA levels >4 $\log_{10}$ copies per ml of plasma and genetic analysis of HIV-1 envelope by conventional clonal sequencing.

[b] Log_{10} HIV-1 RNA copies per ml of plasma by measuring HIV-1 *gag* gene (COBAS® AMPLICOR HIV-1 MONITOR Test, v1.5, Roche, Pleasanton, CA, USA).

[c] Log_{10} HIV-1 *gag* copies/10^6 $CD4^+$ T cells

[d] CD4% is used to normalize for the age-related lymphocytosis that occurs in pediatric subjects.
