# Supplementary material for: High-resolution deep sequencing reveals biodiversity, population structure, and persistence of HIV-1 quasispecies within host ecosystems
Source: Retrovirology. 2012 Dec 17;9:108. doi: 10.1186/1742-4690-9-108 (PMC3531307; doi:10.1186/1742-4690-9-108)
Supplement: Additional file 2 — Error correction. [file 1742-4690-9-108-S2.pdf]

Additional file 2: Error correction.

A two-step error correction strategy included:

1. Correct frame-shifting mutation. Frame shifts reflected insertions and deletions introduced at the read level by the detection algorithm in the Roche/454 process. As V3 has essentially no length variation within an individual, frame-shifting reads were corrected. Sequences were aligned using Geneious (Biomatters Ltd, Auckland, New Zealand) followed by BioEdit (Informer Technologies, Inc). All insertions were removed by alignment using the reference sequence HIV-1_{HXB2} as a mask in BioEdit. Nucleotide sequences were translated into amino acid sequences to define codons; any amino acid codon with one missing nucleotide residue was corrected with the correspondent nucleotide in the reference sequence. Any read with more than two nucleotide deletions within a codon were removed from further analysis.

2. Correct random mismatches. Random mismatches resulted in synonymous or nonsynonymous substitutions or STOP codons. To correct errors resulted from random nucleotide substitutions, Hierarchical clustering of sequences at 3% pairwise distance was performed and a consensus sequence for each cluster was developed using ESPRIT program [25]. Since the rate of mismatch was 0.3% (3 mismatches per 1,000 nt) and length of V3 loop is about 100 bp, only 1 of 3 reads would display a mismatch. The most frequent nucleotide at each position appears in the consensus sequence, thus a low frequency error will not contribute to a consensus sequence. For sequence clusters contain a single sequence (a singleton), there is a chance that 1 of 3 singleton clusters contain mismatch(s), thus people should be cautious when draw any conclusion to results based on singleton sequence clusters.
