# Supplementary material for: High-resolution deep sequencing reveals biodiversity, population structure, and persistence of HIV-1 quasispecies within host ecosystems
Source: Retrovirology. 2012 Dec 17;9:108. doi: 10.1186/1742-4690-9-108 (PMC3531307; doi:10.1186/1742-4690-9-108)
Supplement: Additional file 3 — Table S2. Sequential filtering of data sets through the bioinformatics pipeline. [file 1742-4690-9-108-S3.pdf]

Additional file 3: Table S2. Sequential filtering of data sets through the bioinformatics pipeline.

PID[a]	Raw reads	Reads removed by filters (%)		Error-corrected consensus clusters (no.)	Final reads	
		Quality control	Error correction		%	Number
S1	9,964	6.3	0.5[b]	283	93.2	9,291
S2	9,305	2.8	0.6[c]	282	96.6	8,988
S3	10,238	5.4	5.0	75	89.8	9,194
S4	21,348	3.1	4.9	202	92.1	19,662
S5	10,971	7.1	0.2	172	93.0	10,174
S6	10,599	4.7	0.3	179	95.0	10,067

[a] Patient identification.

[b] Error correction included removal of recombinants found in five unique reads.

[c] Error correction included removal of hypermutated sequences found in about 0.3% of reads.
