# Supplementary material for: High-resolution deep sequencing reveals biodiversity, population structure, and persistence of HIV-1 quasispecies within host ecosystems
Source: Retrovirology. 2012 Dec 17;9:108. doi: 10.1186/1742-4690-9-108 (PMC3531307; doi:10.1186/1742-4690-9-108)

Additional file 4: Figure S1. Phylogenetic tree of clustered error-corrected pyrosequences from individuals studied.

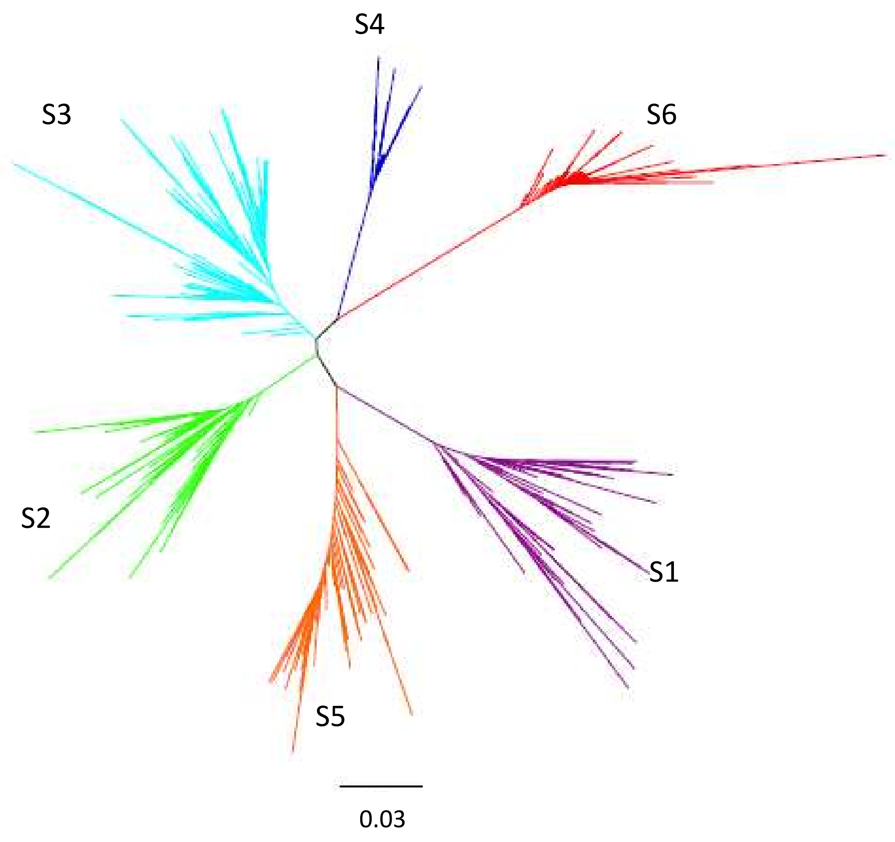

Supplement: Additional file 4 — Figure S1. Phylogenetic tree of clustered error-corrected pyrosequences from individuals studied. [file 1742-4690-9-108-S4.pdf]
