# Supplementary material for: High-resolution deep sequencing reveals biodiversity, population structure, and persistence of HIV-1 quasispecies within host ecosystems
Source: Retrovirology. 2012 Dec 17;9:108. doi: 10.1186/1742-4690-9-108 (PMC3531307; doi:10.1186/1742-4690-9-108)
Supplement: Additional file 5 — Figure S2. Likelihood mapping analysis to evaluate phylogenetic signal. [file 1742-4690-9-108-S5.pdf]

Additional file 5: Figure S2. Likelihood mapping analysis to evaluate phylogenetic signal.

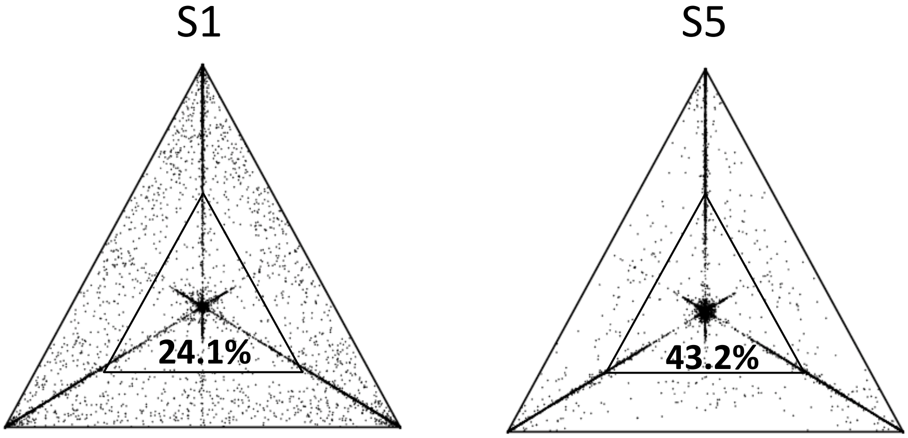

Pinv	5.7%	13.9%

Likelihood mapping analyses were performed with the program TREE-PUZZLE [40] for each data set by analyzing 10,000 random quartets. Star-like signals in the likelihood maps were <45%, indicating sufficient phylogenetic signal [41]. Neither data set showed significant saturation (P<0.0001) [42], indicating that data could be used for reliable phylogeny inferences.
